# Biliary type-II sphincter of Oddi dysfunction with a pancreatic duct dilation: a case report and review of the literature

**DOI:** 10.1186/s13256-021-02674-z

**Published:** 2021-02-22

**Authors:** Naseem Al-Khoury, Okbah Mohamad, Abd Al-Jawad Mazloum, Maher Madi

**Affiliations:** 1 Faculty of Medicine, Tishreen University, Latakia, Syria; 2 Humboldt University, Berlin, Germany; 3 Department of Gastroenterology, Tishreen University Hospital, Latakia, Syria

**Keywords:** Sphincter of Oddi dysfunction, Double-duct sign, Background

## Abstract

**Background:**

The double-duct sign is defined as dilation of both the common bile duct and pancreatic duct, which usually indicates pancreatic malignancy. However, benign causes have also been reported to cause a double-duct sign.

**Case presentation:**

We present the case of a 59-year-old Caucasian female patient admitted to the Gastroenterology Department with intermittent right epigastric abdominal pain and an intact gallbladder. A double-duct sign was seen on endoscopic ultrasound. The suspicion of pancreatic malignancy was excluded through follow-up investigations. Biliary type II sphincter of Oddi dysfunction was diagnosed with an association of the double-duct sign. Sphincterotomy was performed to reduce pain, and there was no recurrence of symptoms during follow-up.

**Conclusions:**

This is the third reported case in the literature of the double-duct sign associated with sphincter of Oddi dysfunction. This case emphasizes that the double-duct sign is not always caused by a local malignancy. The literature review of the reported cases has been summarized to help in the diagnosis of future similar cases.

The double-duct sign presents as dilation of the pancreatic duct and common bile duct on imaging studies. This sign is considered menacing since the two most common causes are malignancies of either the pancreas or the ampulla of Vater [1]. However, benign causes have also been reported to cause a double-duct sign, including chronic pancreatitis and ampullary stenosis [1]. Only two cases in the literature have described a double-duct sign due to sphincter of Oddi dysfunction (SOD) as the underlying etiology [2, 3]. We report the case of a 59-year-old female who presented with abdominal pain and was diagnosed with an unusual cause of the double-duct sign. Also, we performed a literature search on the reported cases of double-duct sign.

## Case presentation

A 59-year-old Caucasian female presented to the Gastroenterology Department for moderate and intermittent upper right quadrant pain of 3-month duration. The pain was not associated with food intake or radiating to other regions. She did not have nausea, vomiting, weight loss, loss of appetite, or a change in bowel habits. The stool and urine colors were also normal.

The patient’s history consisted of osteoporosis, tension headache, chronic recurrent urinary infection, and hypertension. The patient does not smoke or drink alcohol. Her drug history consisted of valsartan and acetaminophen.

On physical examination, vital signs were within the normal range. There was no presence of jaundice, pallor, or edema. Murphy’s sign was negative. (To check for Murphy's sign, the patient is asked to inspire deeply while the examiner palpates the right subcostal region. If pain is elicited and the patient suddenly ceases his/her inspiratory effort, a positive Murphy’s sign has been induced.) Laboratory tests revealed no abnormal results (Table 1).

**Table 1:** Laboratory blood tests

Test	Result	Normal value
Red blood cells	4.63 × 10^9^/l	3.80–5.20 × 10^9^/l
C-reactive protein	3 mg/l	Up to 6 mg/l
Erythrocyte sedimentation rate	10 mm/1 hour	Up to 20 mm/1 hour
Leucocytes	5.7 × 10^9^/l	4–10.5 × 10^9^/l
Conjugated bilirubin	0.8 ml/dl	0.2–1.2 mg/dl
Alkaline phosphatase	88 U/l	26–117 U/l
Gamma-glutamyl transferase	9 U/l	Up to 38 U/l
Alanine aminotransferase	11 U/l	Up to 41 U/l
Aspartate aminotransferase	17 U/l	Up to 40 U/l
Glucose	80 mg/dl	100 mg/dl
Creatinine	0.69 mg/dl	0.9–1.2 mg/dl
Calcium Amylase	8.6 mg/dl 59 U/l	8–10 mg/dl < 100 U/l
CA-19.9	29.93 U/ml	0–37 U/ml

Abdominal computed tomography (CT) scan excluded any obstructive lesion that might have caused the double-duct sign. The abdominal ultrasound showed normal gallbladder.

**Fig. 1.**
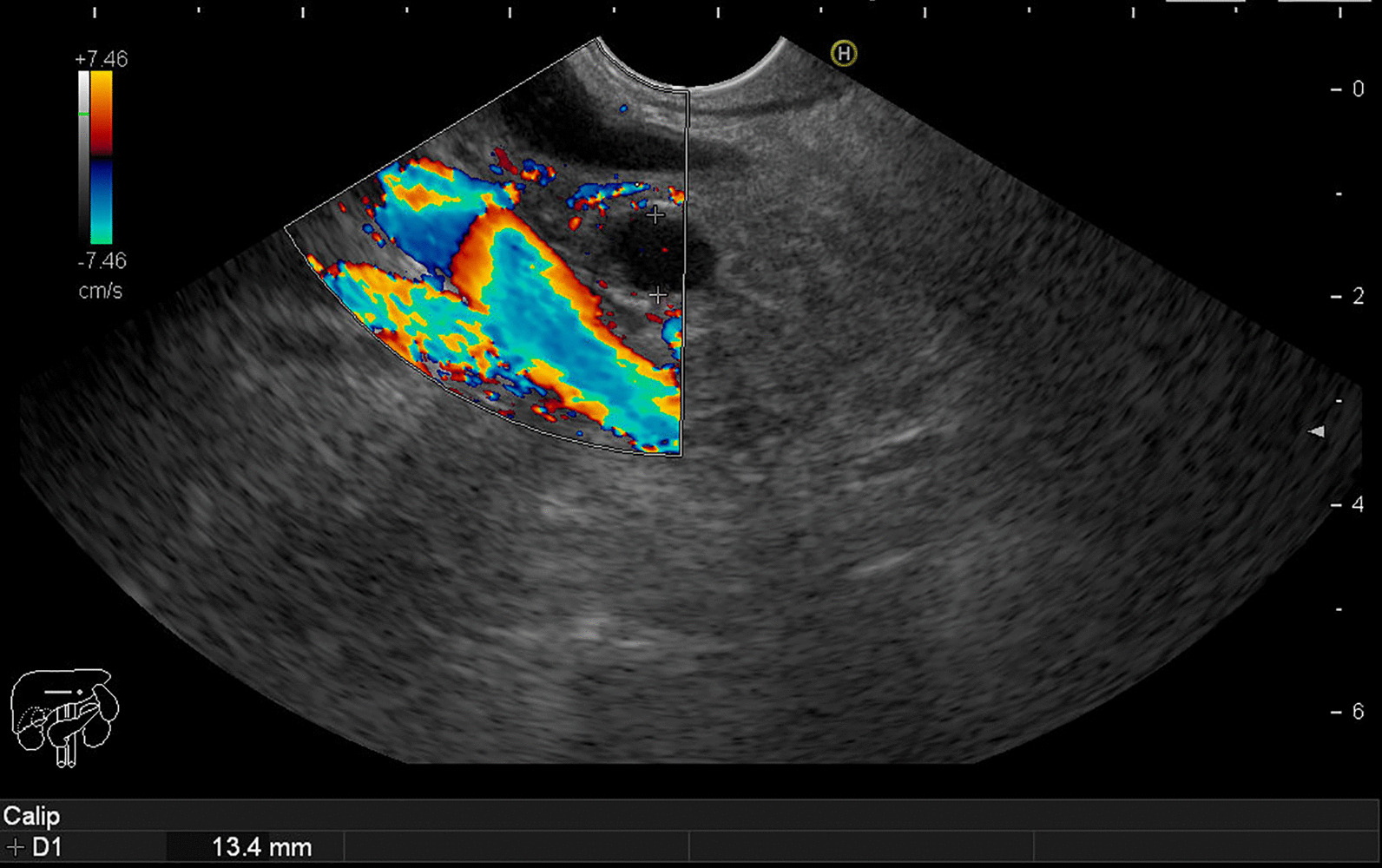
Endoscopic ultrasound showing a dilated common bile duct

**Fig. 2.**
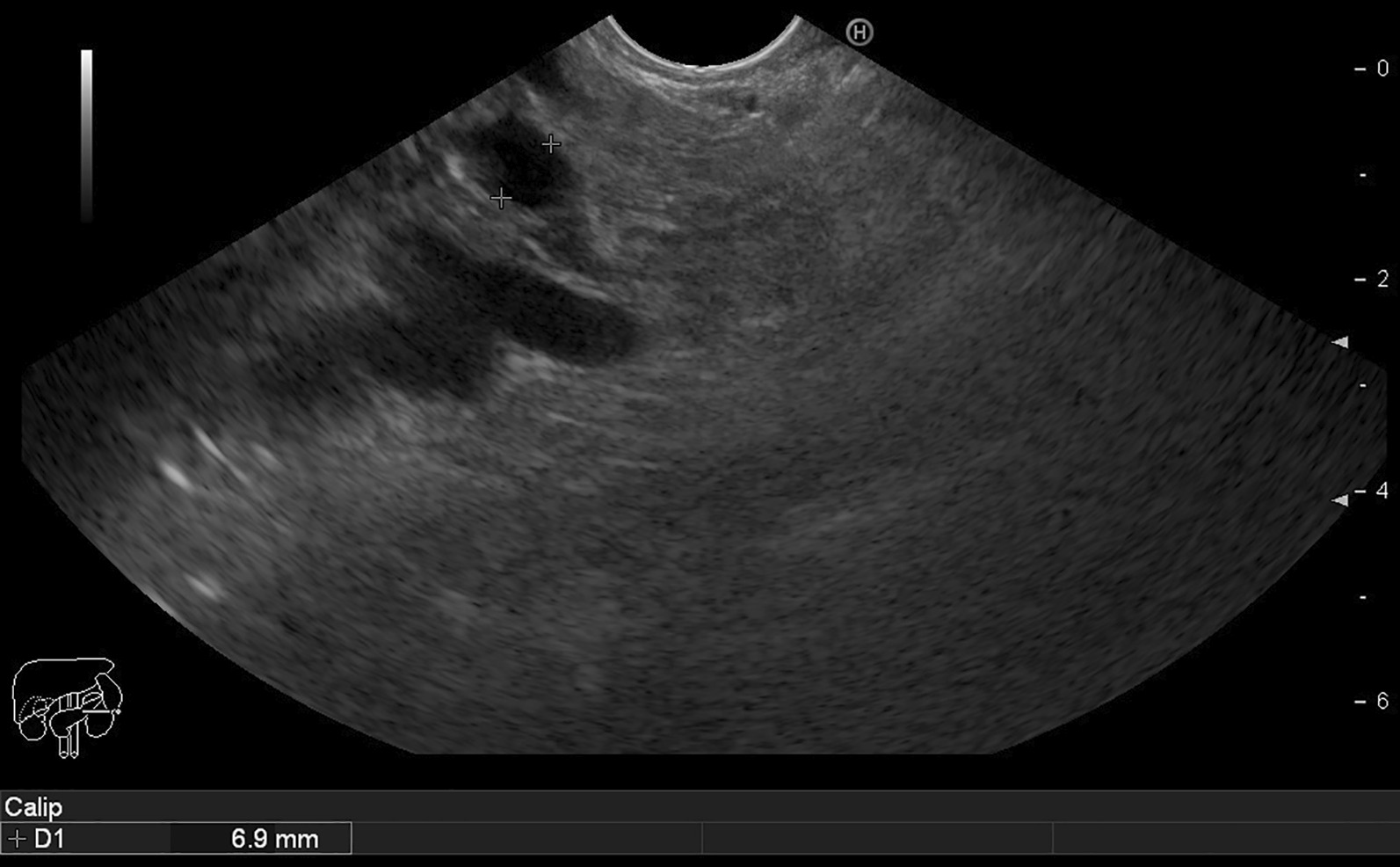
Endoscopic ultrasound showing a dilated pancreatic duct

Endoscopic ultrasound (EUS) showed dilation of the common bile duct (CBD) (13.4 mm) (Fig. 1) and pancreatic duct (PD) (6.9 mm) (Fig. 2) near the head of the pancreas with no stones in the CBD or suspicious mass lesions in the pancreas or the ampulla of Vater.

Endoscopic retrograde cholangiopancreatography (ERCP) confirmed the dilatation of the CBD and PD starting at the level of the ampulla of Vater. The patient’s history and results of applied investigations together with the Milwaukee Classification system established a diagnosis of biliary type II SOD.

During ERCP, a 6-mm endoscopic sphincterotomy was performed with a 7-French plastic stent placement of 5 cm length in the PD followed by a 10-French plastic stent of 9 cm length in the CBD. The pain diminished after sphincterotomy, and the stents were removed after 3 months.

**Fig. 3.**
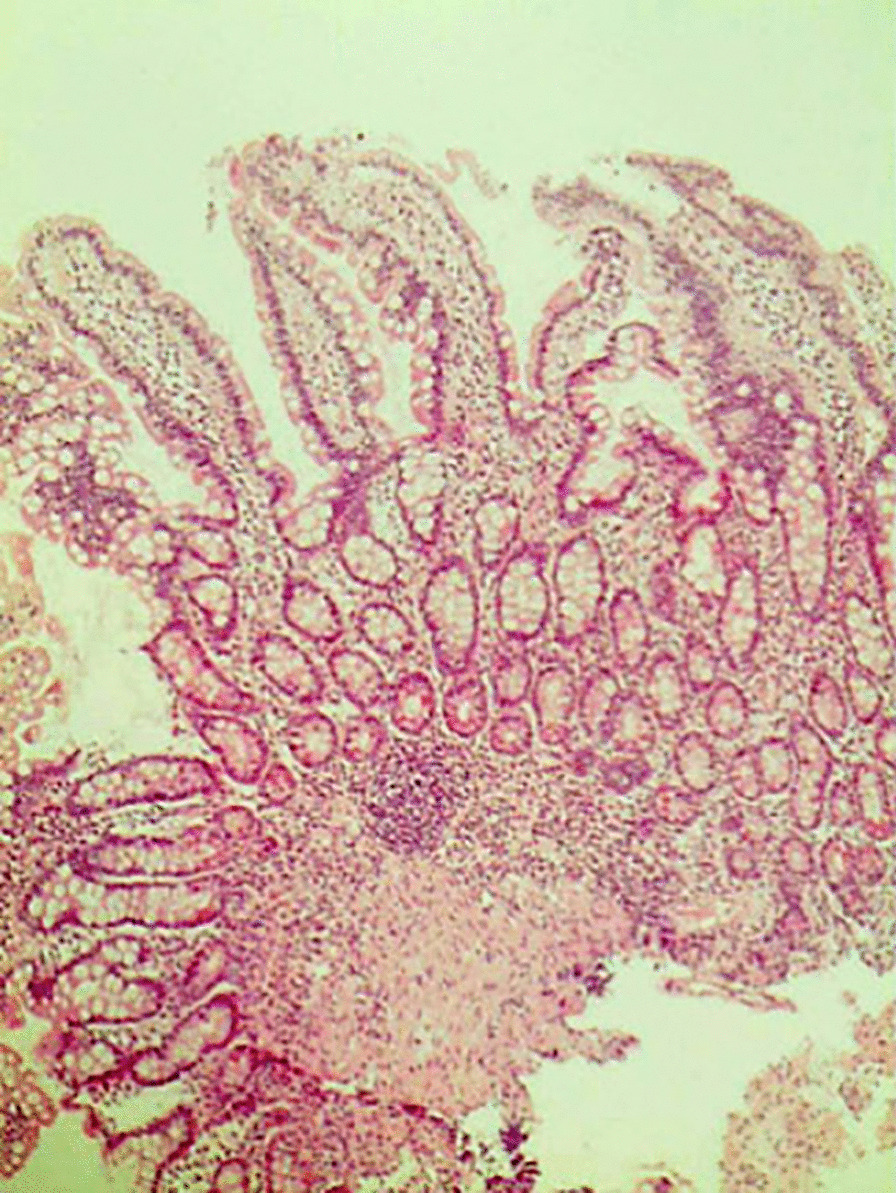
Biopsy of the ampulla of Vater

Biopsy of the ampulla of Vater (Fig. 3) was performed during the ERCP and revealed a mild, chronic, and non-specific inflammation with no atypical cells. After 6 months of follow-up, the patient had a good status with no abnormalities.

## Discussion

Sphincter of Oddi dysfunction is a clinical syndrome characterized by an outflow reduction of bile and pancreatic secretions due to dyskinesia or anatomical stenosis. It has multiple clinical features that may or may not be simultaneous: episodic pain in the epigastrium or the right upper quadrant, nausea, vomiting, and recurrent bouts of pancreatitis [4].

In our case, depending on the patient's history and performed investigations, biliary type II SOD—according to the Milwaukee classification system—was diagnosed [5]. This is defined as a biliary type pain plus a dilated common bile duct > 12 mm on ultrasonography or liver enzyme dysfunction. However, there was no evidence of pancreatic-type pain or pancreatitis features to be classified into the pancreatic type SOD. However, pancreatic duct dilation was present along with common bile duct dilation appearing as a double-duct sign on imaging studies.

SOD is common in women after cholecystectomy [6] and is considered a distinct clinical entity when associated with opium abuse [7].

Our patient had not undergone cholecystectomy, had no exposure to opiate use, and did not have evidence of gallstone disease. SOD was of uncertain origin in this case. To our knowledge, no previous cases in the literature have reported the double-duct sign as a result of biliary type II SOD with a pancreatic duct dilation without the presence of pancreatitis. Two cases of double-duct sign were due to opiate-associated sphincter of Oddi dysfunction [2, 3]. The double-duct sign usually indicates pancreatic malignancy with a lower prevalence in patients who do not have obstructive jaundice [8]. In academic literature, other rare cases of the double-duct sign have been reported. We performed a PubMed search in July 2020 with 'double-duct sign' as search term. There were 77 publications, 8 of which were excluded because the articles were not in English; 53 included original data, and 16 reports are summarized according to the main cause that led to the double-duct sign in Table 2.

**Table 2:** Case reports of double-duct sign

Condition	References
Strongyloides stercoralis	[9]
Brunner’s gland hamartoma	[10]
Serous cystadenoma	[11]
Pancreas carcinoma not seen on abdominal ultrasound or CT scan	[12]
Gallstones	[8]
Somatostatinoma	[13]
Sphincter of Oddi dysfunction	[2, 3]
Pancreaticobiliary ascariasis	[14]
IgG4-related disease mimicking pancreatic cancer	[15]
Ampullary cancer demonstrating Courvoisier's sign	[16]
Right Bochdalek hernia with right liver agenesis	[17]
Afferent loop syndrome after pylorus-preserving pancreaticoduodenectomy	[18]
Gastroduodenal intussusception (GISTs)	[19]
Carcinoma of Vater's ampulla	[20]
Carcinoma of the pancreas associated with anomalous junction of pancreaticobiliary tracts	[21]

CT: Computed tomography, Gists: Gastrointestinal stromal tumors, IgG4: Immunoglobulin G4

Since pancreatic cancer grows and develops in a stealthy fashion, early detection and diagnosis with possible therapeutic resection are challenging [1]. Hence, it is important to exclude malignancies when a double-duct sign is seen. After exclusion, we should seek for other possible causes, including the rare ones.

## Conclusion

In our patient, sphincter of Oddi dysfunction was the underlying cause for the double-duct sign. To our knowledge, this is the third description of a double-duct sign due to sphincter of Oddi dysfunction. Our case emphasizes that a double-duct sign is not always caused by a local malignancy. Nevertheless, a cancer of the pancreas or ampulla of Vater should be ruled out as early as possible.

## Data Availability

Data mentioned in this case report are available to the reviewers if required.

## Abbreviations

SOD: Sphincter of Oddi dysfunction
CBD: Common bile duct
PD: Pancreatic duct
ERCP: Endoscopic retrograde cholangiopancreatography
